# Supplementary material for: Global Performance of Human Papillomavirus Typing, Screening, and Evaluation As Assessed Using Proficiency Panels Traceable to International Standards: A Retrospective Analysis, 2008–2024
Source: J Med Virol. 2026 Jun 29;98(7):e71033. doi: 10.1002/jmv.71033 (PMC13312892; doi:10.1002/jmv.71033)
Supplement: Supplementary file 1 — Table S1: Proficiency of detecting HPV types by laboratories that participated in 2024 PP, with data from 2008, 2010, 2011, 2013, 2014, 2017, 2019, 2021, 2022 and 2023. Laboratories were classified according to the proportion of correct HPV type identifications across the full panel: 100% proficient, 99–90% proficient, 89–80% proficient, < 80% proficient, or not proficient (did not meet the minimum proficiency criteria for the panel year). Values are shown as number of laboratories/total laboratories tested (%). Table S2: Two most commonly reported assays by genotyping proficiency study year. The table shows the two most frequently reported assays in each proficiency study year, expressed as the number of datasets using each assay out of the total number of submitted datasets for that year, with percentages. [file JMV-98-e71033-s001.docx]

**Supplementary Table 1:** Proficiency of detecting HPV types by laboratories that participated in 2024 PP, with data from 2008, 2010, 2011, 2013, 2014, 2017, 2019, 2021, 2022 and 2023.

| **Proficiency** | **All test by laboratories that participated in**  **2008, 2010, 2011, 2013, 2014, 2017, 2019, 2021, 2022, 2023 and 2024** | | | | | | | | | | |
| --- | --- | --- | --- | --- | --- | --- | --- | --- | --- | --- | --- |
|  | **2008 (%)** | **2010 (%)** | **2011 (%)** | **2013 (%)** | **2014 (%)** | **2017 (%)** | **2019 (%)** | **2021 (%)** | **2022 (%)** | **2023**  **(%)** | **2024**  **(%)** |
| **100 % proficient** | 3 / 11  (27) | 3 / 16  (19) | 12 / 25  (48) | 9 / 24  (37) | 11 / 26  (42) | 19 / 41  (46) | 10 / 39  (26) | 74 / 95  (78) | 80 / 97  (82) | 49 / 64  (77) | 101 / 128  (79) |
| **99-90 % proficient** | 1 / 11  (9.1) | 1 / 16  (6.2) | 1 / 25  (4.0) | 2 / 24  (8.3) | 2 / 26  (7.7) | 4 / 41  (9.8) | 10 / 39  (26) | 1 / 95  (1.1) | 3 / 97  (3.1) | 3 / 64  (4.7) | 10 / 128  (7.8) |
| **89-80 % proficient** | 0 / 11  (0) | 3 / 16  (19) | 2 / 25  (8.0) | 4 / 24  (17) | 3 / 26  (12) | 4 / 41  (9.8) | 2 / 39  (5.1) | 0 / 95  (0) | 0 / 97  (0) | 1 / 64  (1.6) | 1 / 128  (0.8) |
| **<80 % proficient** | 2 / 11  (18) | 1 / 16  (6.2) | 2 / 25  (8.0) | 0 / 24  (0) | 0 / 26  (0) | 0 / 41  (0) | 1 / 39  (2.6) | 0 / 95  (0) | 1 / 97  (1.0) | 1 / 64  (1.6) | 2 / 128  (1.6) |
| **Not proficient** | 5 / 11  (45) | 8 / 16  (50) | 8 / 25  (32) | 9 / 24  (37) | 10 / 26  (38) | 14 / 41  (34) | 16 / 39  (41) | 20 / 95  (21) | 13 / 97  (13) | 10 / 64  (16) | 14 / 128  (11) |

Laboratories were classified according to the proportion of correct HPV type identifications across the full panel: 100% proficient, 99–90% proficient, 89–80% proficient, <80% proficient, or not proficient (did not meet the minimum proficiency criteria for the panel year). Values are shown as number of laboratories / total laboratories tested (%).

**Supplementary Table 2**. Two most commonly reported assays by genotyping proficiency study year.

| **Year** | **Most common assay** | **Datasets, n/N (%)** | **Second most common assay** | **Datasets, n/N (%)** |
| --- | --- | --- | --- | --- |
| 2008 | Linear Array (Roche) | 11/136 (8.09) | PapillomaFinder (Pathofinder) | 10/136 (7.35) |
| 2010 | Linear Array (Roche) | 17/132 (12.88) | InnoLiPA (Innogenetics) | 12/132 (9.09) |
| 2011 | Linear Array (Roche) | 18/134 (13.43) | In-house PCR Luminex | 11/134 (8.21) |
| 2013 | Linear Array (Roche) | 11/136 (8.09) | PapillomaFinder (Pathofinder) | 10/136 (7.35) |
| 2014 | Linear Array (Roche) | 14/148 (9.46) | HPV Direct Flow-chip (Master Diagnostica) | 14/148 (9.46) |
| 2017 | HPV Direct Flow-chip (Master Diagnostica) | 18/141 (12.77) | Linear Array (Roche) 11 10 | 13/141 (9.22) |
| 2019 | Anyplex II HPV 28 (Seegene) | 20/110 (18.18) | In-house PCR Luminex | 7/110 (6.36) |
| 2021 | Hybribio 21 array HPV (Hybribio) | 29/211 (13.74) | Anyplex II HPV 28 (Seegene) | 21/211 (9.95) |
| 2022 | Hybribio 37 array HPV (Hybribio) | 21/154 (13.64) | HPV-23 Genotyping (Hybriobio) | 20/154 (12.99) |
| 2023 | Allplex HPV28 (Seegene) | 14/142 (9.86) | Hybribio 21 array HPV (Hybribio) | 14/142 (9.86) |
| 2024 | Hybribio 37 array HPV (Hybribio) | 21/169 (12.43) | Hybribio 21 array HPV (Hybribio) | 20/169 (11.83) |

The table shows the two most frequently reported assays in each proficiency study year, expressed as the number of datasets using each assay out of the total number of submitted datasets for that year, with percentages.
